# The Conjugated Bile Acids Profile Suggests a Novel Liver‐Muscle Axis Associated With Sarcopenia in Chronic Liver Disease

**DOI:** 10.1111/liv.70612

**Published:** 2026-03-28

**Authors:** Motoh Iwasa, Akiko Eguchi, Motoyuki Kohjima, Teruo Miyazaki, Hiroshi Kitamura, Yuko Takami, Naoki Yamashita, Mina Tempaku, Kiyora Izuoka, Yoshinao Kobayashi, Yoshiyuki Takei, Akira Honda, Hayato Nakagawa, Tadashi Ikegami, Makoto Nakamuta, Jun Okabe, Aldo J. Montano‐Loza

**Affiliations:** ^1^ Department of Gastroenterology and Hepatology Mie University Graduate School of Medicine Tsu Japan; ^2^ Department of Gastroenterology Murase Hospital Suzuka Japan; ^3^ Biobank Center Mie University Hospital Tsu Japan; ^4^ Department of Gastroenterology Clinical Research Institute, NHO Kyushu Medical Center Fukuoka Japan; ^5^ Joint Research Center Tokyo Medical University, Ibaraki Medical Center Inashiki‐gun Japan; ^6^ Department of Laboratory Animal Medicine Tohoku University School of Medicine Sendai Japan; ^7^ Department of Hepato‐Biliary‐Pancreatic Surgery NHO Kyushu Medical Center Fukuoka Japan; ^8^ Department of Gastroenterology and Hepatology Tokyo Medical University, Ibaraki Medical Center Inashiki‐gun Japan; ^9^ Epigenetics in Human Health and Disease Laboratory Baker Heart and Diabetes Institute Melbourne Victoria Australia; ^10^ Division of Gastroenterology and Liver Unit University of Alberta Edmonton Canada

**Keywords:** conjugated bile acids, humoral factor, liver cirrhosis, muscle fibre type shift, organ cross‐talk, sarcopenia, skeletal muscle inflammation

## Abstract

**Background:**

Liver‐related sarcopenia is a devastating systemic complication of chronic liver disease (CLD) driven by mechanisms extending beyond nutritional deficiency. However, the role of liver‐derived humoral factors remains unclear. We utilised a unique cohort of human skeletal muscle biopsies to test the hypothesis that serum conjugated bile acids (C‐BAs) act as key mediators of this liver‐muscle cross‐talk.

**Methods:**

Serum and *rectus abdominis* muscle samples were meticulously collected from 36 CLD patients and 6 non‐CLD controls during elective surgery. Multifidus‐erector spinae and psoas muscle areas were quantified from CT images. Comprehensive correlations were analysed between C‐BAs and molecular markers of muscle inflammation and fibre‐type composition. These findings were supplemented by in vitro validation using GCDCA treatment of C2C12 myotubes.

**Results:**

Serum C‐BAs levels were significantly elevated in CLD patients. The liver cirrhosis (LC) group exhibited a significantly smaller multifidus‐erector spinae area (32.06 ± 8.05 cm^2^) compared to controls (39.78 ± 4.38 cm^2^, *p* = 0.019). Muscle area loss was strongly correlated with hepatic reserve deterioration (ALBI/ALB) and systemic inflammation (serum IL‐6). Crucially, muscle area was negatively correlated with the ratio of tauro‐C‐BAs (*p* < 0.05). Muscle biopsies showed a molecular shift: enrichment of the slow‐twitch fibre marker myosin‐heavy chain 7 (MYH7, type I) and a concomitant reduction of the fast‐twitch marker MYH4 (type IIb), alongside signs of local chronic inflammation (*p* < 0.05). The reduction in MYH4 was correlated with glyco‐C‐BAs, a finding replicated in GCDCA‐treated C2C12 myotubes.

**Conclusions:**

Elevated C‐BAs may represent a critical, liver‐derived humoral factor associated with the pathological features of liver‐related sarcopenia. C‐BA‐associated muscle mass loss and systemic inflammation are reflected at the molecular level by a shift toward a slow‐twitch phenotype, accumulation of macrophages and altered energy metabolism in muscle biopsies. These findings suggest that C‐BAs may serve as a potentially actionable therapeutic target for mitigating muscle catabolism and improving clinical outcomes in CLD patients.

## Lay Summary

- Elevated conjugated bile acids (C‐BAs) are suggested to be a liver‐derived humoral factor that is associated with reduced muscle mass and muscle pathology in human chronic liver disease (CLD).
- C‐BAs elevation is associated with detrimental molecular and phenotypic reprogramming of muscle.
- C‐BAs may represent an actionable therapeutic target. Modulating BA profiles could offer a strategy for risk stratification and for mitigating muscle catabolism in vulnerable CLD patients.

**Abbreviations**
γ‐GT: glutamyl‐transferase
AGER: the specific receptor for advanced glycation end‐product
AIF1: allograft inflammatory factor 1
ALB: albumin
ALBI: albumin‐bilirubin
ALP: alkaline phosphatase
ALT: alanine aminotransferase
AST: aspartate aminotransferase
BA: bile acid
CA: cholic acid
CCL2: C‐C motif chemokine ligand 2
CDCA: chenodeoxycholic acid
CH: chronic hepatitis
CLD: chronic liver disease
CLEC10A: c‐type lectin domain containing 10A
DCA: deoxycholic acid
IL‐6: interleukin‐6
LC: liver cirrhosis
MYH: myosin‐heavy chain
PAX7: paired box 7
PEPCK: phosphoenolpyruvate carboxykinase
PLT: platelet count
PT: prothrombin time
SERPINE1: serpin family E member 1
SREBF1: sterol regulatory element binding transcription factor 1
T‐Bil: total bilirubin
TFAM: mitochondrial transcription factor A
TGFB1: transforming growth factor beta 1
TNF: tumour necrosis factor

## 1 Introduction

Sarcopenia is a progressive and generalised skeletal muscle disorder characterised by a decline in muscle volume and strength [1]. It is highly prevalent in older adults and patients with cancer or chronic disease, causing detrimental effects on clinical outcomes such as falls, fractures, physical disability and mortality [2, 3]. The prevalence of sarcopenia in patients with chronic liver disease (CLD) is higher than in other chronic diseases [4]. Previous studies have suggested that the causes of sarcopenia in liver cirrhosis (LC) include low serum branched‐chain amino acid (BCAA) levels [5], decreased serum vitamin D levels [6], abnormal insulin growth factor‐1 (IGF‐1) and increased reactive oxygen species and increased inflammatory cytokines [7]. Several signalling pathways, including ubiquitin‐proteasome degradation, mammalian target of rapamycin (mTOR) inhibition and myostatin activation [8], are involved in the pathogenesis of sarcopenia in LC. However, the molecular mechanisms underpinning the liver‐muscle axis involved in CLD‐related sarcopenia are not fully understood [9]. Understanding muscle pathophysiology unique to CLD is essential for the development of effective interventions to protect muscle mass and function.

We previously reported that lithocholic acid (LCA) acts as a positive regulator of skeletal muscle mass in CLD rats and mediates skeletal muscle cell hypertrophy in differentiated C2C12 myotubes [10]. However, human data are still lacking regarding the role of bile acids (BAs) as potential regulators of skeletal muscle originating in the liver that would indicate a liver‐muscle axis. Recent studies have shown that tauro‐ and glyco‐conjugated BAs (C‐BAs) are involved in the pathogenesis of metabolic syndrome and dementia [11, 12]. In addition, it has been reported that elevated concentrations of BAs significantly contribute to liver fibrosis and portal hypertension [13, 14], and tauro‐C‐BAs correlated with serum proinflammatory markers and hepatic inflammatory pathways [15]. We also reported that C‐BAs were associated with liver damage and survival in human and rat CLDs, but not with hepatocellular carcinoma (HCC) [16]. This evidence enabled us to analyse the association of C‐BAs with muscle changes using precious abdominal muscle samples obtained during surgery for HCC in the present study. Furthermore, circulating levels of pro‐inflammatory cytokines, such as interleukin (IL)‐6, tumour necrosis factor‐α (TNF‐α) and IL‐1β, have been associated with sarcopenia and physical frailty [17].

Human skeletal muscle is mainly composed of type I fibres, which are oxidative with slow contraction speed adapted for aerobic work, and type II fibres, which are glycolytic, have fast contraction speed, and confer strength. Type I and IIa muscle fibres contain high levels of mitochondrial oxidative phosphorylation enzymes and low levels of glycolytic enzymes, whereas type IIb shows the opposite enzyme profile [18]. In age‐associated sarcopenia, accelerated loss of motor units and fibre atrophy are primarily observed in type II fibres, while the proportion of type I fibres tends to increase [19, 20]. In the context of illness/disease, several reports indicate that the reduction in the proportion of type I fibre occurring in the lower limb muscle of patients with chronic obstructive pulmonary disease is associated with disease severity [21]. However, no study exists concerning the relationship between muscle loss and a shift in muscle fibre type in patients with CLD; thus, we hypothesise that a part of BAs and/or IL‐6 may be involved in the pathological change in the muscle.

We aim to explore the mechanisms underlying the effect of BAs and chronic inflammation on skeletal muscle fiber type profile and muscle mass in patients with CLD.

## 2 Patients and Methods

### 2.1 Patient Cohort

Prior to the initiation of the study, the protocol was reviewed and approved by the clinical research ethics review committee of NHO Kyushu Medical Center (Approval No. 10–29) and informed written consent was obtained from all patients before enrollment. In addition, this study was performed retrospectively on stored samples using Approval No. 10–29, and patients can opt out of their data being used, which was approved by the clinical research ethics review committee of Mie University Hospital (Approval No. H2020‐035) and NHO Kyushu Medical Center (Approval No. 20C039). Thirty‐six HCC patients undergoing partial hepatectomy and 6 patients with other abdominal malignancies (non‐CLD), including liver metastasis from colorectal cancer (*n* = 2) or breast cancer (*n* = 1), pancreatic cancer (*n* = 1), gallbladder cancer (*n* = 1) and common bile duct cancer (*n* = 1) were enrolled in this study from August 2010 to March 2014. The diagnosis for HCC and other abdominal malignancies was based on histological examination obtained during surgery. Chronic hepatitis (CH) or LC was diagnosed through histological evaluation following hepatectomy. The psoas and multifidus‐erector spinae muscle area at the middle of the third lumbar vertebra (L3) (cm^2^) was manually measured from computed tomography (CT) images using SliceOmatic (Canada). Thresholds of −29 to +150 Hounsfield Units (HU) were used to anatomically delineate the psoas and multifidus‐erector spinae muscle index [area (cm^2^) and then were divided by height (m^2^)] to calculate muscle indexes [22].

### 2.2 Clinical and Laboratory Assessments

Clinical data at the time of surgery included anthropometric measures, and laboratory parameters, including aspartate aminotransferase (AST), alanine aminotransferase (ALT), gamma‐glutamyltransferase (γ‐GT), alkaline phosphatase (ALP), albumin (ALB), total bilirubin (T‐Bil), prothrombin time (PT) and platelet count (PLT), were retrospectively evaluated. The albumin‐bilirubin (ALBI) score was calculated from serum ALB and T‐Bil levels [23]. Blood samples were collected before surgery and kept at −80°C until BAs composition and IL‐6 measurement. The level of IL‐6 was quantified using commercially available ELISA kits (R&D Systems, Minneapolis, MN, USA) following the manufacturer's instructions.

BA concentration was determined blindly as described by Ando et al. [24], with minor modifications. After the addition of internal standards and 0.5 M potassium phosphate buffer (pH 7.4), BAs were extracted using Bond Elut C18 cartridges and quantified by Liquid Chromatograph‐Mass Spectrometry (LC–MS)/MS. Chromatographic separation was performed using a Hypersil GOLD column (150 × 2.1 mm, 3.0 μm; Thermo Fisher Scientific) at 40°C. The mobile phase consisted of (i) 20 mM ammonium acetate buffer (pH 7.5)‐acetonitrile‐methanol (70:15:15, v/v/v) and (ii) 20 mM ammonium acetate buffer (pH 7.5)‐acetonitrile‐methanol (30:35:35, v/v/v). The following gradient programme was used at a flow rate of 200 μL/min: 0–100% B for 20 min, hold 100% B for 10 min and re‐equilibrate to 100% A for 8 min. The concentrations of primary BAs, secondary BAs, 12α‐hydroxysterol (12α‐OH) BAs and non‐12α‐OH BAs were calculated based on the sum of the concentrations of each type of BA.

### 2.3 Muscle Biopsies Analysis

**FIGURE 1:**
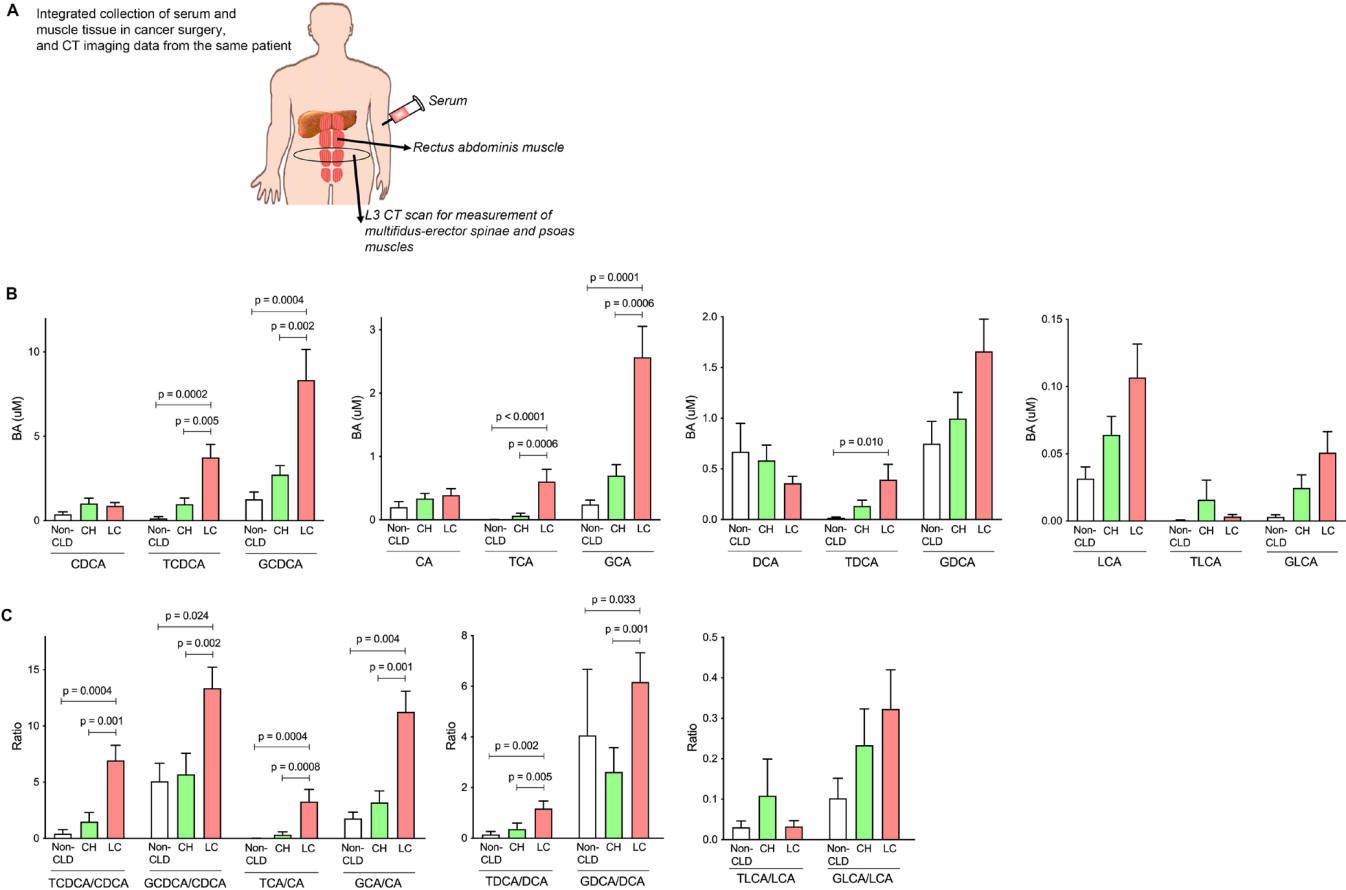
Changes in BAs among the three groups. (A) The overview of this study. (B) Changes in CDCA, CA, DCA and LCA and their conjugates among the three groups. (C) Changes in the ratio of conjugated to unconjugated CDCA, CA, DCA and LCA among the three groups. BA, bile acid; CA, cholic acid; CDCA, chenodeoxycholic acid; CH, chronic hepatitis; CLD, chronic liver disease; DCA, deoxycholic acid; LC, liver cirrhosis; LCA, lithocholic acid. *p < 0.05; **p < 0.01; ***p < 0.001; ****p < 0.0001.

A biopsy specimen was obtained from the *rectus abdominis* muscle at the time of surgery. After making an incision in the skin and dissecting through the subcutaneous fat, the anterior sheet of the *rectus abdominis* muscle was opened with scissors, and a muscle biopsy specimen weighing approximately 0.5 g was obtained. The biopsy specimen was immediately frozen in liquid nitrogen and then stored at −80°C until analysis. No complications occurred from the biopsy procedure. The overview of this study is illustrated in Figure 1A.

### 2.4 Immunohistochemistry

*Rectus abdominis* muscle sections (10 μm) were prepared from frozen samples, and then the slides were stored at −80°C until analysis. Immunohistochemistry staining for myosin‐heavy chain (MYH) 7 (Type I) and MYH2 (Type IIa) (BA‐F8 and SC‐71, respectively; Developmental Studies Hybridoma Bank, Iowa, IA, USA) was performed in frozen sections using a secondary antibody (MP‐7714, Vector Laboratories, Newark, CA, USA) according to the manufacturer's instructions. All pictures were taken using an Olympus CKX53 (Olympus, Japan).

### 2.5 Cell Culture and Treatment

Mouse myoblasts C2C12 cells were maintained in Dulbecco's Modified Eagle Medium (DMEM) containing 20% foetal bovine serum at 37°C and 5% CO2. The confluent cells were differentiated into myotubes by culturing with DMEM containing 2% horse serum and glycochenodeoxycholic acid (GCDCA) (Millipore‐Sigma, Japan). The medium, supplemented with GCDCA, was changed daily.

### 2.6 Gene Expression

Total RNA was isolated from the *rectus abdominis* muscle using TRI Reagent (Molecular Research Center, Cincinnati, OH, USA) according to the manufacturer's instructions. The cDNA was synthesised from total RNA using a cDNA Synthesis kit (Takara, Shiga, Japan). Real‐time PCR quantification was performed using the SYBR Green PCR mixture (Thermo Fisher Scientific Inc) and the Quantstudio1 (Thermo Fisher Scientific Inc). The PCR primers for muscle fibre types, metabolism and inflammation used to amplify each gene are listed in Table S1. Mean values of mRNA were normalised to beta 2 microglobulin (β2m).

### 2.7 Statistical Analyses

Continuous variables are presented as median (25th and 75th percentiles), and categorical variables are shown as numbers of patients. Continuous data were compared using the Mann–Whitney *U*, the *t*‐test for two groups or the Kruskal–Wallis in multiple groups. Categorical data were compared using the Chi‐squared test. The relationship between serum BA levels and clinical data was examined using Spearman's rank correlation coefficient. Multivariable linear regression analyses were performed to evaluate the independent associations between LC and skeletal muscle area, muscle index and *MYH* expression. Age, sex and body mass index (BMI) were included as covariates a priori as potential confounders. Regression coefficients (β), 95% confidence intervals (CIs) and *p* values were reported. All statistical analyses were performed using SPSS23.0 software (IBM, Armonk, NY, USA) and Prism 9 (GraphPad Software Inc., CA, USA). Differences were considered significant at *p*‐value < 0.05.

## 3 Results

### 3.1 Patient Characterisation

In this study, 36 patients with CLD and HCC patients with Barcelona Clinic Liver Cancer (BCLC) stage 0/A and six patients with other abdominal malignancies were included. Patients with CLD were categorised into the CH and the LC groups. Table S2 presents a comparison of baseline clinical characteristics and laboratory variables between groups. The CH group (11 men and 3 women with a median age of 79.5 [74.5 and 81.3] years) and the LC group (6 men and 16 women with a median age of 74.0 [66.8 and 78.0] years). Patients with CH were older (*p* = 0.015) and had a higher frequency of males compared to the LC group (*p* = 0.005). Patients with LC had a higher BMI compared to non‐CLD (*p* = 0.007).

Viral causative agents accounted for 5 HBV and 31 HCV cases. In the LC group, AST (*p* < 0.001), ALT (*p* = 0.032), ALP (*p* = 0.004) and T‐Bil (*p* = 0.003) levels were significantly higher, while the ALB (*p* < 0.001), ALBI score (*p* < 0.0001) and PLT (*p* = 0.018) were significantly lower when compared to the CH group. The non‐CLD group included six cases of abdominal malignancies other than HCC, without accompanying CLD. All subjects in this study had cancer without cachexia (Table S2).

### 3.2 Conjugated BAs Were Significantly Elevated in CLD Patients

The total BA (LC vs. non‐CLD: *p* < 0.001 or vs. CH: *p* = 0.010), primary BA (LC vs. non‐CLD: *p* < 0.001 or vs. CH: *p* = 0.003), secondary BA (LC vs. non‐CLD: *p* = 0.002), 12α‐OH BA (LC vs. non‐CLD: *p* = 0.004 or vs. CH: *p* = 0.007) and non‐12α‐OH BA (LC vs. non‐CLD: *p* < 0.001 or vs. CH: *p* = 0.027 and non‐CLD vs. CH: *p* = 0.044) levels were significantly higher in the LC group than in the non‐CLD or CH group (Figure S1, Tables S3 and S4). Additionally, tauro‐ and glyco‐conjugated chenodeoxycholic acids (TCDCA [LC vs. non‐CLD: *p* < 0.001 or vs. CH: *p* = 0.005] and GCDCA [LC vs. non‐CLD: *p* < 0.001 or vs. CH: *p* = 0.002]) and cholic acids (TCA [LC vs. non‐CLD: *p* < 0.001 or vs. CH: *p* < 0.001] and GCA [LC vs. non‐CLD: *p* < 0.001 or vs. CH: *p* < 0.001]) were significantly higher in the LC group than in the non‐CLD or CH group, whereas unconjugated CA and CDCA showed no significant difference (Figure 1B and Table S4). Moreover, the ratio of conjugated to unconjugated BAs, such as TCDCA/CDCA (LC vs. non‐CLD: *p* < 0.001 or vs. CH: *p* = 0.001), GCDCA/CDCA (LC vs. non‐CLD: *p* = 0.024 or vs. CH: *p* = 0.002), TCA/CA (LC vs. non‐CLD: *p* < 0.001 or vs. CH: *p* < 0.001) and GCA/CA (LC vs. non‐CLD: *p* = 0.004 or vs. CH: *p* = 0.001) were significantly higher in the LC group than in the non‐CLD or CH group (Figure 1C). Although deoxycholic acid (DCA) showed no significant difference (Figure 1B), the ratios to their free forms (TDCA/DCA [LC vs. non‐CLD: *p* = 0.002 or vs. CH: *p* = 0.005] and GDCA/DCA [LC vs. non‐CLD: *p* = 0.033 or vs. CH: *p* = 0.001]) were significantly higher in the LC group than in the non‐CLD or CH group (Figure 1C and Table S4). There was no significant difference among the three groups in terms of LCA (Figure 1B,C). The changes observed in these CLD cases were consistent with previous reports [25, 26].

### 3.3 Correlation Between the Ratios of Conjugated to Unconjugated BAs and Laboratory Variables

TCDCA/CDCA, GCDCA/CDCA, TCA/CA, GCA/CA and GDCA/DCA ratios exhibited negative correlations with ALB, PT and PLT, with positive correlations observed in AST, T‐Bil and ALBI scores. GCDCA/CDCA exhibited a negative correlation with PLT and a positive correlation with AST, ALT, γ‐GT and T‐Bil (Table S5). These results show that the ratio of tauro‐ and glyco‐conjugated to unconjugated BAs increased concomitantly with the progression of CLD.

### 3.4 Muscle Area Shows a Significant Negative Correlation, Whereas Serum IL‐6 Levels Show a Significant Positive Correlation, With the Ratio of Tauro‐Conjugated to Unconjugated BAs

**FIGURE 2:**
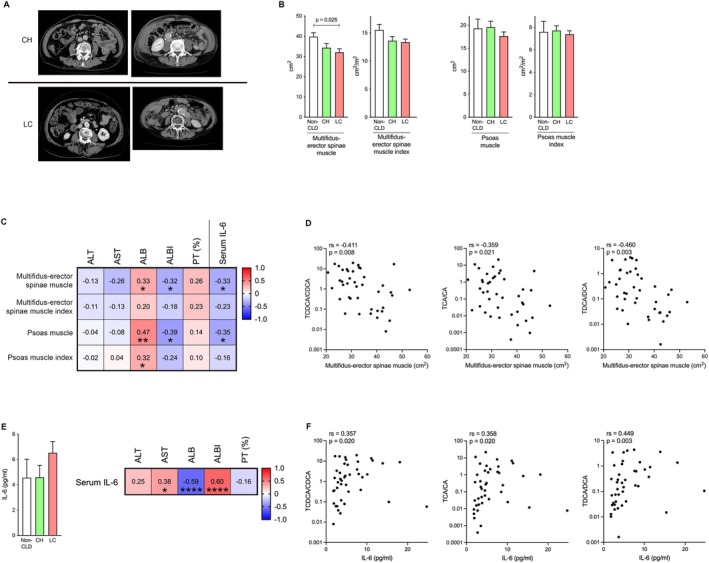
Changes in skeletal muscle area/index and their correlation with laboratory variables. (A) Axial CT images from patients with CH and LC. (B) Changes in multifidus‐erector spinae muscle area and index among the three groups. Changes in psoas muscle area and index among the three groups. (C) Correlation of multifidus‐erector spinae and psoas muscle area/index with laboratory variables and IL‐6. (D) Scatter plot between multifidus‐erector spinae muscle area and the ratio of conjugated to unconjugated BA. (E) Changes in IL‐6 among the three groups. Correlation of IL‐6 with laboratory variables. (F) Scatter plot between IL‐6 and the ratio of conjugated to unconjugated BA. ALB, albumin; ALBI, albumin‐bilirubin; ALT, alanine aminotransferase; AST, aspartate aminotransferase; CA, cholic acid; CDCA, chenodeoxycholic acid; CH, chronic hepatitis; CLD, chronic liver disease; CT, computed tomography; DCA, deoxycholic acid; IL‐6, interleukin‐6; LC, liver cirrhosis; PT, prothrombin time. Positive correlations are shown with varying shades of red, whereas negative correlations are represented with varying shades of blue, according to their strength. The values in the figure represent the correlation coefficients, and * indicates statistical significance. *p < 0.05; **p < 0.01; ****p < 0.0001.

Figure 2A shows axial CT images from representative patients with CH and LC. Statistical analysis revealed that the multifidus‐erector spinae muscle area (cm^2^) was significantly diminished in the LC group compared to the non‐CLD group (*p* = 0.025) (Figure 2B). A trend‐level difference was observed in the psoas muscle area (Figure 2B). In the multivariable analysis, age and sex were identified as significant predictors of muscle area, whereas the independent effect of CLD was less pronounced in this small cohort (Table 1). Multifidus‐erector spinae and psoas muscle areas showed significant correlations with liver function test values such as ALB (rs = 0.33, *p* < 0.05; rs = 0.47, *p* < 0.01) and ALBI score (rs = −0.32, *p* < 0.05; rs = −0.39, *p* < 0.05), as well as significant correlations with IL‐6 (rs = −0.33, *p* < 0.05; rs = −0.35, *p* < 0.05) (Figure 2C). In addition, the psoas muscle index was also correlated with ALB (rs = 0.32, *p* < 0.05) (Figure 2C). Notably, a negative correlation was observed between the multifidus‐erector spinae muscle area and TCDCA/CDCA (rs = −0.411, *p* = 0.008), TCA/CA (rs = −0.359, *p* = 0.021) and TDCA/DCA (rs = −0.460, *p* = 0.003) (Figure 2D). Additionally, significant correlations were observed between tauro‐C‐BAs and multifidus‐erector spinae muscle area (Figure S2A), multifidus‐erector spinae muscle index (Figure S2B) and serum IL‐6 levels (Figure S2C).

**TABLE 1:** Multivariable linear regression analysis for muscle area or muscle fiber types.

Variables	Estimate (95% CI profile likelihood)	|*t*|	*p*
Model 1: Multifidus‐erector spinae muscle area			
β1 CH = 0, LC = 1	1.541 (−3.565 to 6.647)	0.6155	0.5427
β2 Age	−0.3717 (−0.6701 to −0.0734)	2.541	0.0163^a^
β3 Sex (Male = 1)	9.797 (4.931 to 14.66)	4.106	0.0003^a^
β4 BMI	−0.555 (−1.222 to 0.1116)	1.698	0.0995
Model 2: Psoas muscle area			
β1 CH = 0, LC = 1	−0.1297 (−3.354 to 3.095)	0.082	0.9325
β2 Age	−0.1701 (−0.3585 to 0.0182)	1.842	0.075
β3 Sex (Male = 1)	4.715 (1.643 to 7.787)	3.13	0.0038^a^
β4 BMI	−0.1596 (−0.5805 to 0.2613)	0.7735	0.4451
Model 3: Muscle fiber type I (*MYH7*)			
β1 CH = 0, LC = 1	1.218 (−0.2692 to 2.706)	1.681	0.1044
β2 Age	0.01354 (−0.07512 to 0.1022)	0.3134	0.7564
β3 Sex (Male = 1)	0.1277 (−1.297 to 1.553)	0.1839	0.8555
β4 BMI	0.2218 (0.01948 to 0.4242)	2.249	0.0328^a^
Model 4: Muscle fiber type IIx (*MYH1*)			
β1 CH = 0, LC = 1	1.71 (−0.1871 to 3.607)	1.841	0.0756
β2 Age	−0.0542 (−0.1644 to 0.0559)	1.006	0.3227
β3 Sex (Male = 1)	0.907 (−0.8923 to 2.706)	1.029	0.3115
β4 BMI	0.0719 (−0.1757 to 0.3195)	0.593	0.5576
Model 5: Muscle fiber type IIb (*MYH4*)			
β1 CH = 0, LC = 1	−0.0436 (−0.0776 to −0.0097)	2.628	0.0136^a^
β2 Age	0.0003 (−0.0016 to 0.0023)	0.3512	0.728
β3 Sex (Male = 1)	−0.0127 (−0.04490 to 0.0195)	0.8059	0.4269
β4 BMI	−0.0033 (−0.0077 to 0.0012)	1.493	0.1462
Model 6: Muscle fiber type IIa (*MYH2*)			
β1 CH = 0, LC = 1	5.369 (−6.343 to 17.08)	0.9362	0.3567
β2 Age	−0.5353 (−1.215 to 0.1446)	1.608	0.1183
β3 Sex (Male = 1)	13.66 (2.555 to 24.77)	2.512	0.0176^a^
β4 BMI	0.4219 (−1.106 to 1.950)	0.5638	0.5771

Abbreviations: BMI, body mass index; CH, chronic hepatitis; CI, confidence interval; LC, liver cirrhosis; MYH, myosin‐heavy chain.

^a^ indicates statistical significance.

Several groups had already reported that serum IL‐6 levels were elevated in CLD [27]. Indeed, serum IL‐6 levels showed a tendency to be higher in the LC group compared to non‐CLD and CH (Figure 2E). Serum IL‐6 levels show a significant correlation with liver function test values such as AST (rs = 0.38, *p* < 0.05), ALB (rs = −0.59, *p* < 0.0001) and ALBI score (rs = 0.60, *p* < 0.0001), as we expected (Figure 2E). Furthermore, serum IL‐6 levels also significantly correlated with the ratio of tauro‐conjugated to unconjugated BAs (TCDCA/CDCA [rs = 0.357, *p* = 0.020], TCA/CA [rs = 0.358, *p* = 0.020] and TDCA/DCA [rs = 0.449, *p* = 0.003]) (Figure 2F). Skeletal muscle mass loss is associated with reduced hepatic functional reserve and elevated serum IL‐6 levels. Notably, the ratio of tauro‐conjugated to unconjugated BAs correlates with both muscle volume and serum IL‐6 levels, suggesting that an imbalance in BA metabolism may be involved in the process of muscle catabolism.

### 3.5 Muscle Fibre Types and Correlation With Local Chronic Inflammation and Energy Metabolism

**FIGURE 3:**
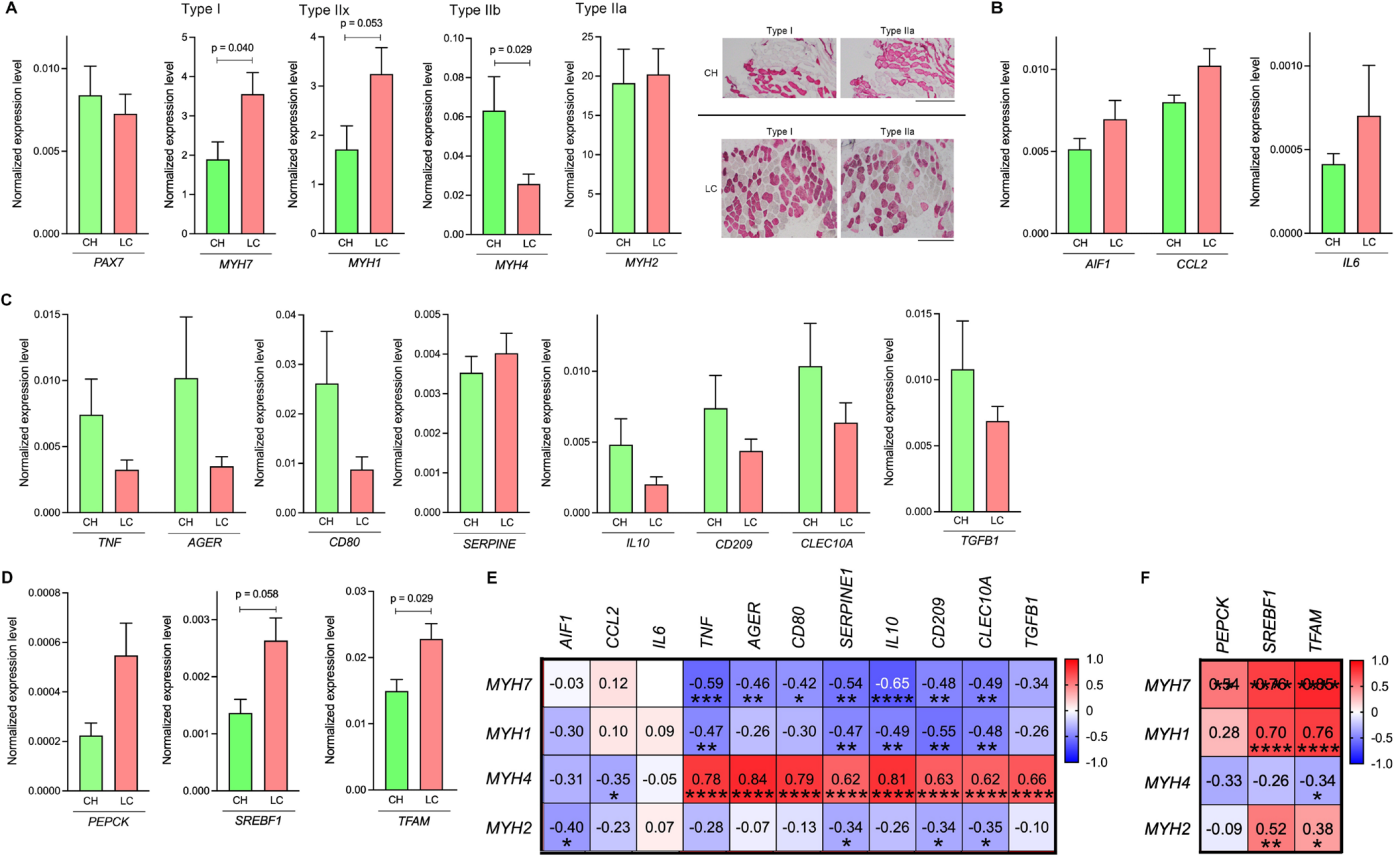
Comparison of muscle fibre types, metabolic changes and inflammation in rectus abdominis muscle between CH and LC, and correlation of gene expression. (A) Gene expression of myogenic marker and muscle fibre types in rectus abdominis muscle between CH and LC. Immunohistochemical staining for MYH7 (type I) and MYH2 (type IIa) in rectus abdominis muscle sections from one patient with CH and LC. Scale bar, 500 μm. (B) Gene expression of inflammation‐related genes in rectus abdominis muscle between CH and LC. (C) Gene expression of pro‐ and anti‐inflammatory genes in rectus abdominis muscle between CH and LC. (D) Gene expression of metabolic‐related genes in rectus abdominis muscle between CH and LC. (E) Correlation of inflammation‐related and pro‐ and anti‐inflammatory genes with muscle fibre type gene expression. (F) Correlation of metabolic‐related genes with muscle fibre type gene expression. AGER, the specific receptor for advanced glycation end‐product; AIF1, allograft inflammatory factor 1; CCL2, C‐C motif chemokine ligand 2; CH, chronic hepatitis; CLEC10A, c‐type lectin domain containing 10A; IL‐6, interleukin‐6; LC, liver cirrhosis; MYH, myosin heavy chain; PAX7, paired box 7; PEPCK, phosphoenolpyruvate carboxykinase; SERPINE1, serpin family E member 1; SREBF1, sterol regulatory element binding transcription factor 1; TFAM, mitochondrial transcription factor A; TGFB1, transforming growth factor beta 1; TNF, tumour necrosis factor. Positive correlations are shown with varying shades of red, whereas negative correlations are represented with varying shades of blue, according to their strength. The values in the figure represent the correlation coefficients. *p < 0.05; **p < 0.01; ***p < 0.001; ****p < 0.0001.

The correlation of multifidus‐erector spinae and psoas muscle area with liver function, as well as BA composition, led us to investigate the pathophysiological changes of the muscle. The mRNA level of *paired box 7* (*PAX7*), a gene linked with development and regeneration, was slightly decreased in the LC group (Figure 3A). Muscle fibre type was different in the LC group; the mRNA levels of *MYH7* for type I (slow) (*p* = 0.040) and *MYH1* for type IIx (fast) (*p* = 0.053) were increased, whereas *MYH4* mRNA levels for type IIb (very fast) were significantly lower in the muscle of LC patients compared to the CH group (*p* = 0.029) (Figure 3A). The major fibre type in the *rectus abdominis* muscle, *MYH2* for type IIa (intermediate), was decreased in CLD patients but was not changed between the CH and LC (Figure 3A). In consecutive sections of the *rectus abdominis* muscle from one representative patient, an increase in *MYH7* expression for type I was also observed in the LC group compared to the CH group. In contrast, *MYH2* expression for type IIa was stably expressed in both groups (Figure 3A). Multivariable linear regression analysis revealed that the presence of LC was independently associated with reduced *MYH4* expression (*β* = −0.044, 95% CI −0.078 to −0.010, *p* = 0.0136), even after adjusting for age, sex and BMI (Table 1). No significant associations were observed between *MYH4* expression and age, sex or BMI. In addition, none of the other MYH isoforms showed a significant association with the progression of CLD (Table 1).

Next, we investigated inflammation in the *rectus abdominis* muscle. The mRNA levels of *allograft inflammatory factor 1* (*AIF1*), *C‐C motif chemokine ligand 2* (*CCL2*) and *IL6* tended to be elevated in patients with LC (Figure 3B), suggesting the accumulation of macrophages in LC patients. Furthermore, both pro‐ and anti‐inflammatory genes, including *tumour necrotic factor* (*TNF*), *receptor for advanced glycation end‐product specific receptor* (*AGER*), *CD80, IL10*, *CD209*, *c‐type lectin domain containing 10A* (*CLEC10A*) and *transforming growth factor beta 1* (*TGFB1*), were altered in LC patients indicating perturbation of local inflammation in the skeletal muscle (Figure 3C). We further explored metabolic changes in the rectus abdominis muscle, including gluconeogenesis‐related processes, lipid synthesis and mitochondrial function. Recent studies show that skeletal muscle has a certain gluconeogenic capacity and that sterol regulatory element binding transcription factor 1 (SREBF1) levels respond to nutritional status in skeletal muscle [28, 29]. The mRNA levels of *phosphoenolpyruvate carboxykinase* (*PEPCK*), *SREBF1* (*p* = 0.058) and *mitochondrial transcription factor A* (*TFAM*) (*p* = 0.029) were increased in the LC group compared to the CH group (Figure 3D). The mRNA levels of *ALF1* and *CCL2* were not associated with fibre types, except for a negative correlation of *ALF1* with *MYH2* (rs = −0.40, *p* < 0.05) and *CCL2* with *MYH4* (rs = −0.35, *p* < 0.05) (Figure 3E). Notably, the changes of inflammation (pro‐and anti‐inflammatory genes)‐related genes were negatively correlated with type I (*MYH7* vs. *TNF* [rs = −0.59, *p* < 0.001], vs. *AGER* [rs = −0.46, *p* < 0.01], vs. *CD80* [rs = −0.42, *p* < 0.05] vs. *SERPINE1* [rs = −0.54, *p* < 0.01], vs. *IL10* [rs = −0.65, *p* < 0.0001], vs. *CD209* [rs = −0.48, *p* < 0.01] or vs. *CLEC10A* [rs = −0.49 *p* < 0.01]) and were positively correlated with type IIb (*MYH4* vs. *TNF* [rs = 0.78, *p* < 0.0001], vs. *AGER* [rs = 0.84, *p* < 0.0001], vs. *CD80* [rs = 0.79, *p* < 0.0001], vs. *SERPINE1* [rs = 0.62, *p* < 0.0001], vs. *IL10* [rs = 0.81, *p* < 0.0001], vs. *CD209* [rs = 0.63, *p* < 0.0001], vs. *CLEC10A* [rs = 0.62, *p* < 0.0001] or vs. *TGFB1* [rs = 0.66, *p* < 0.0001]) (Figure 3E). On the other hand, these inflammation‐related gene expressions showed little association with the clinical variables (Figure S3). Additionally, the metabolic changes in *PEPCK*, *SREBF1* and *TFAM* were significantly and positively correlated with changes in fibre types, such as type I, IIx and IIa (*PEPCK* vs. *MYH7* [rs = 0.54, *p* < 0.01]; *SREBF1* vs. *MYH7* [rs = 0.76, *p* < 0.0001], vs. *MYH1* [rs = 0.70, *p* < 0.0001] or vs. *MYH2* [rs = 0.52, *p* < 0.01]; *TFAM* vs. *MYH7* [rs = 0.85, *p* < 0.0001], vs. *MYH1* [rs = 0.76, *p* < 0.0001] or vs. *MYH2* [rs = 0.38, *p* < 0.05]) (Figure 3F). In contrast, these metabolic changes tended to correlate negatively with changes in type IIb (*MYH4*, rs = −0.34, *p* < 0.05) (Figure 3F). These results indicated that changes in muscle fibre types were associated with modulation in energy metabolism and local chronic inflammation.

### 3.6 Fibre Type Shifts, Macrophage Infiltration and Energy Metabolism in the Rectus Abdominis Muscle Correlate With Liver Dysfunction

**FIGURE 4:**
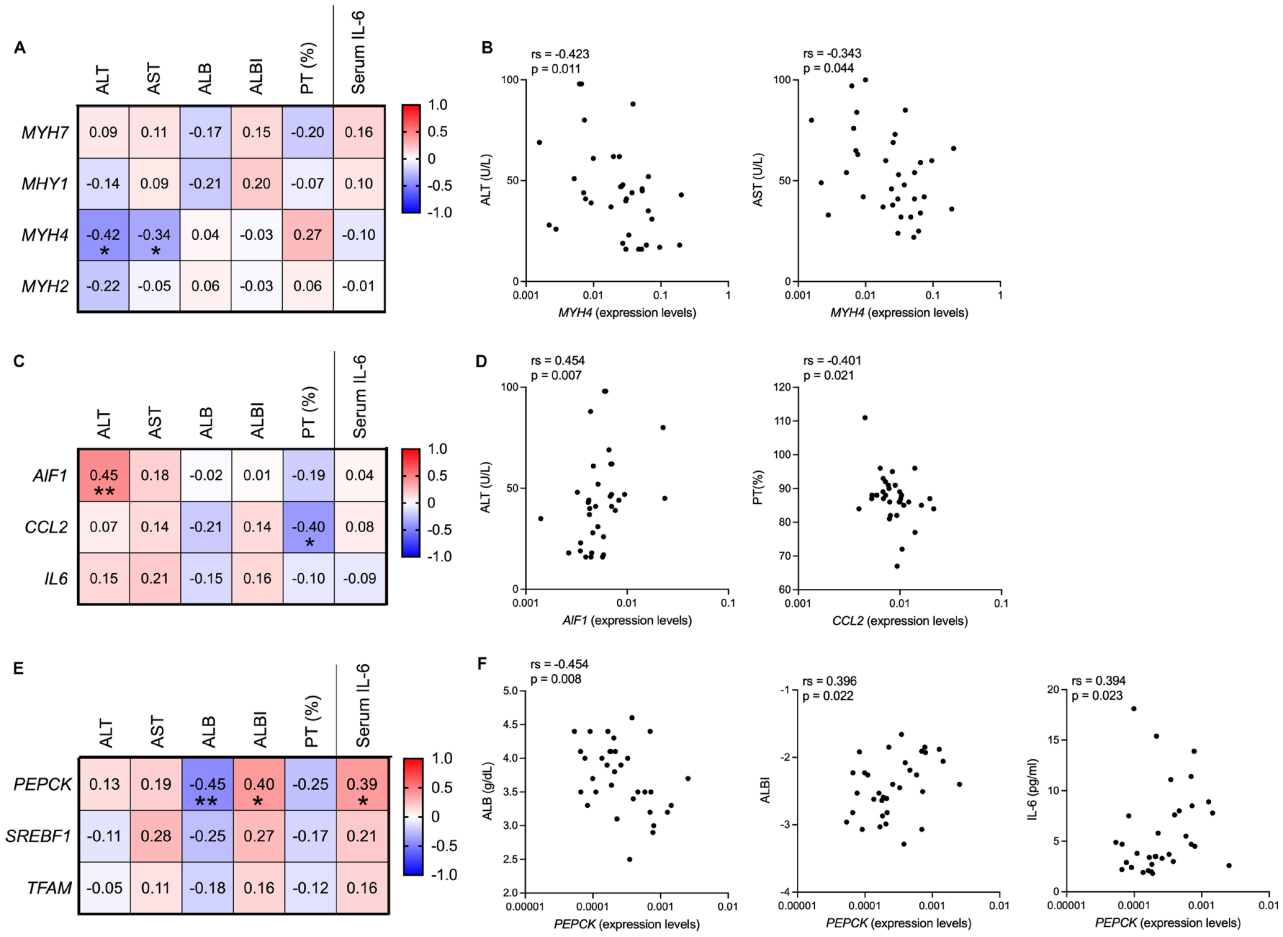
Correlation between muscle gene expressions in abdominal muscles and clinical variables and serum IL‐6 levels. (A, B) Muscle fibre types and clinical variables. Correlations (A) and scatter plots (B) of muscle gene expressions of fibre types (*MYH7*, *MYH1*, *MYH4* and *MYH2*) against laboratory variables for liver function and serum IL‐6 levels. (C, D) Macrophage infiltration gene expression and clinical variables. Correlations (C) and scatter plot (D) of macrophage infiltration (*ALF1* and *CCL2*) and *IL6* genes against laboratory variables for liver function and serum IL‐6 levels. (E, F) Energy metabolism gene expressions and clinical variables. Correlations (E) and scatter plot (F) of energy metabolism genes (*PEPCK*, *SREBF1* and *TFAM*) against laboratory variables for liver function and serum IL‐6 levels. AIF1, allograft inflammatory factor 1; ALB, albumin; ALBI, albumin–bilirubin; ALT, alanine aminotransferase; AST, aspartate aminotransferase; CCL2, C‐C motif chemokine ligand 2; IL‐6, interleukin‐6; MYH, myosin heavy chain; PEPCK, phosphoenolpyruvate carboxykinase; PT, prothrombin time; SREBF1, sterol regulatory element binding transcription factor 1; TFAM, mitochondrial transcription factor A. Positive correlations are shown with varying shades of red, whereas negative correlations are represented with varying shades of blue, according to their strength. The values in the figure represent the correlation coefficients, and * indicates statistical significance. *p < 0.05; **p < 0.01.

To examine whether LC affects pathophysiological changes in the *rectus abdominis* muscle, we investigated the association of mRNA levels in the *rectus abdominis* muscle with clinical variables, including liver enzymes, hepatic functional reserve and serum IL‐6 levels. For fibre type shifts, mRNA levels of *MYH4* were negatively correlated with liver enzymes ALT (rs = −0.42, *p* < 0.05) and AST (rs = −0.34, *p* < 0.05) (Figure 4A,B). We observed significant correlations such as *AIF1* vs. ALT (rs = 0.45, *p* < 0.01) and *CCL2* vs. PT (rs = −0.40, *p* < 0.05) (Figure 4C,D). Regarding energy metabolism, the mRNA levels of *PEPCK* correlated with clinical markers of liver dysfunction (ALB [rs = −0.45, *p* < 0.01] and ALBI [rs = 0.40, *p* < 0.05]), and IL‐6 (rs = 0.395, *p* < 0.05) (Figure 4E,F). These results show that liver function and IL‐6 are associated with the fibre type shifts, macrophage infiltration and altered energy metabolism within the rectus abdominis muscle.

### 3.7 Conjugated BAs Are Associated With Fibre Type Shifts, Macrophage Infiltration and Altered Energy Metabolism in the Rectus Abdominis Muscle

**FIGURE 5:**
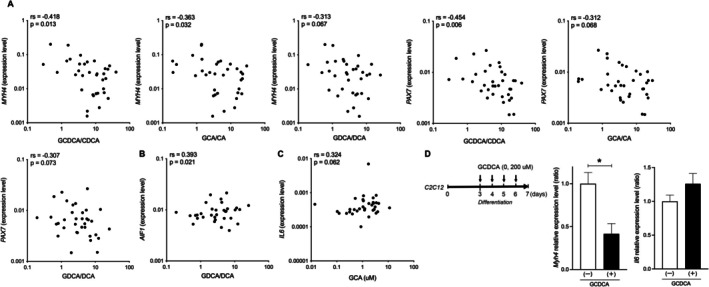
Correlation between the ratio of conjugated to unconjugated BAs or conjugated BAs and muscle gene expression. (A) Correlation between the ratio of conjugated to unconjugated BAs and muscle gene expression, *MYH4* and *PAX7*. (B) Correlation between GDCA/DCA and muscle *AIF1* expression. (C) Correlation between GCA and muscle *IL6* expression. (D) Scheme of experimental design in C2C12 myoblast to myotubes treated with GCDCA and gene expression, *Myh4* and *Il6*, in the presence or absence of GCDCA. AIF1, allograft inflammatory factor 1; CA, cholic acid; CDCA, chenodeoxycholic acid; DCA, deoxycholic acid; IL6, interleukin‐6; MYH, myosin heavy chain; PAX7, paired box 7.

The correlation of liver function and IL‐6 with pathophysiological changes in the *rectus abdominis* muscle led us to further explore the association between BA composition and mRNA levels in the rectus abdominis muscle. Notably, only three correlations were observed between fibre type shifts and C‐BAs (Figure 5A). GCDCA/CDCA was negatively correlated with *MYH4* (rs = −0.418, *p* = 0.013) and *PAX7* (rs = −0.454, *p* = 0.006), while GCA/CA was negatively correlated with *MYH4* (rs = −0.363, *p* = 0.032) mRNA levels (Figure 5A). Regarding macrophage infiltration, GDCA/DCA showed a positive correlation with *AIF1* (rs = 0.393, *p* = 0.021) (Figure 5B), and GCA showed a trend toward a positive correlation with muscular *IL6* mRNA levels (rs = 0.324, *p* = 0.062) (Figure 5C).

We finally explored the direct effects of GCDCA/CDCA, which had the most significant and negative correlation with *MYH4* in the *rectus abdominis* muscle, on muscle fibre type changes in C2C12 myoblasts, which rapidly differentiate to form myotubes. The daily addition of 200 μM of GCDCA led to a significant downregulation of *MYH4* mRNA levels (*p* = 0.015) and upregulation of *IL‐6* mRNA levels in C2C12 cells, indicating the inhibition of type IIb fibre type and the involvement of IL‐6 in this process by GCDCA (Figure 5D). These results revealed that C‐BAs caused by liver dysfunction mediated the muscle damage, including muscle fibre type changes, macrophage infiltration and energy metabolism.

## 4 Discussion

In the present study, we examined abdominal skeletal muscle tissue from patients who underwent surgery for CLD with HCC, or other abdominal malignancies (non‐CLD), to investigate muscle fibre type alterations associated with CLD and the causative underlying mechanisms. For the first time, we demonstrated that elevated levels of tauro‐ and glyco‐C‐BAs affect myosin fibre types and inflammatory states in patients with CLD. We hypothesised that one or more CLD‐related molecules/factors may reduce skeletal muscle mass via a liver‐muscle axis. Indeed, we found that C‐BAs are associated with the progression of CLD, suggesting their potential to induce a transition from fast to slow muscle fiber as well as muscle mass loss.

The initial discovery in the present study revealed a muscle fibre type transition from fast‐twitch (type IIb) to slow‐twitch (type I, which is resistant to fatigue), along with a decline in muscle volume in CLD patients. Our results aligned with a similar study demonstrating that patients with decompensated cirrhosis have a myopathy characterised by fibre type II atrophy and disruption of nuclear positioning [30], which could be related to elevated levels of tauro‐ and glyco‐C‐Bas.

Our multivariable linear regression analysis demonstrated that the presence of LC was independently associated with reduced *MYH4* expression, even after adjusting for potential confounders such as age, sex and BMI. Since MYH4 is a specific marker for fast‐twitch glycolytic fibres, these results suggest that cirrhosis per se induces a distinct shift in muscle fibre composition, independent of the effects of ageing or physical constitution. Muscle fibre fatigue is driven by insufficient cellular metabolism. The increase in slow fibre mass seen in our study was associated with an increase in mitochondrial activity. Moreover, this transition was accompanied by an upregulation of energy metabolism‐related genes. In mice with overexpression of *PEPCK* in skeletal muscle, improved exercise tolerance has been reported [31]. Based on this, the changes in the skeletal muscle of our patients with CLD are presumed to occur as a compensatory response.

Although muscle wasting occurs in a variety of conditions ranging from muscle disuse to cancer cachexia, from ageing sarcopenia to sepsis, the prevalence of sarcopenia in patients with CLD is likely to be higher than what is observed in other diseases [4, 19, 20]. A shift from fast to slow twitch muscle fibres with preferential atrophy of fast glycolytic fibres is observed in cancer cachexia. Selective atrophy of fast fibres has been described in muscle biopsies from human cancer patients [32] and mouse models of cancer cachexia [33]. One of the mechanisms involved in the selective atrophy of fast twitch muscle fibres in cancer cachexia and sepsis is the pro‐inflammatory cytokine TNFα [34, 35, 36]. In LC rats, TNFα levels are increased and correlated with muscle weight and myofiber diameter [7]. Moreover, a recent study observed decreased muscle mass and a shift from fast to slow twitch muscle fibre type in CLD mice [37]. In the present study, macrophage accumulation‐related genes were upregulated, and pro‐ and anti‐inflammatory genes were altered with significant positive or negative correlations with *MYH4* or *MYH7* mRNA levels. Chronic inflammation may be involved in a shift from fast to slow twitch muscle fibre type in the skeletal muscles of patients with CLD.

We found that serum IL‐6 was related to skeletal muscle area, but the gene expression of *IL6* in skeletal muscle was not associated with the expression of *MYH*. In addition, supplementation of GCDCA upregulated *IL6* mRNA levels in C2C12 cells. Based on our data, IL‐6 did not affect muscle fibre type composition by paracrine *signalling*. On the other hand, IL‐6 regulates protein homeostasis in skeletal muscle [38]. While an acute increase in systemic IL‐6 promotes muscle growth and hypertrophy, its sustained elevation, as seen in cancer or sepsis, causes muscle atrophy [39, 40]. IL‐6 is upregulated in LC, contributing to liver cell necrosis and the development of fibrosis [27]. A recent study showed that IL‐6, via the glycoprotein 130/JAK2/STAT3 pathway, mediates sepsis‐induced muscle atrophy, possibly contributing to intensive care unit‐acquired weakness [41]. In our study, serum IL‐6 levels increased in parallel with the progression of liver disease, and muscle area was lower in the LC group, which was associated with serum IL‐6 levels. Although studies in CLD patients have not been reported, these findings are consistent with previous reports describing alterations in skeletal muscle architecture in patients with cancer cachexia and sepsis. It should be noted that our cohort included patients with HCC and metastatic abdominal malignancies; therefore, the presence of malignancy itself may have influenced the presence of sarcopenia. Even in the absence of overt cachexia, malignant tumours may affect systemic inflammation, BA metabolism and skeletal muscle biology. In addition, all CLD patients in this study had early‐stage HCC (BCLC stage 0/A) and were eligible for surgical treatment. Thus, caution is warranted when extrapolating these findings to patients with decompensated cirrhosis, in whom sarcopenia is most clinically relevant. Taken together, while our results suggest an association between CLD progression, serum IL‐6 levels and skeletal muscle alterations, further studies in larger and more diverse cohorts, including patients without malignancy and those with advanced cirrhosis, are needed to better define the independent contribution of CLD to muscle biology.

An important finding of this study was that tauro‐ and glyco‐C‐BAs are critical players in the observed changes in skeletal muscles. In vitro experiments using C2C12 myoblasts also supported these findings. GCA, TCA, GCDCA and TCDCA are significantly altered among different stages of hepatitis B‐induced LC [25]. In addition, higher concentrations of C‐BAs, specifically GCDCA and TCA, are found in the serum of HCC patients compared to healthy controls [26]. Reports suggesting an increase in tauro‐ and glyco‐C‐BAs with the progression of CLD align with our research findings. A recent study showed that dysregulation in BA metabolism, such as an increase in BA conjugation, is involved in the development of cancer cachexia, including muscle loss in mice [42]. Another study reported that tauro‐ and glyco‐C‐BAs induce higher inflammatory responses than free BAs in adipocytes and hepatocytes, which is associated with increased *CCL2* and *IL‐6* expression and increased neutrophil infiltration [43, 44]. BAs can directly disrupt the plasma membrane and activate the protein kinase C (PKC) pathway, which results in an inflammatory response [45]. Indeed, TCA/CA, TCDCA/CDCA and TDCA/DCA showed a negative correlation with muscle volume and a positive correlation with serum IL‐6 levels. GCA/CA was negatively correlated with *MYH4* for type IIb (very fast) mRNA levels and GDCA/DCA was positively correlated with *AIF1* mRNA levels in our study. These results suggest that C‐BAs mediated fibre‐type shifts and macrophage accumulation, leading to a decrease in muscle mass, although we need further study to explore the molecular mechanism.

The results of this study are important, as ileal BA transporter inhibitors (IBATs), like maralixibat and odevixibat, are medications used to treat cholestatic pruritus by reducing BA buildup in the liver, and interestingly have shown improved growth in patients with Alagille syndrome [46].

While our work provides evidence for alterations in skeletal muscle during CLD, several limitations should be acknowledged. The small sample size and the absence of a healthy control group necessitate a cautious interpretation of our findings. In particular, our control group consisted of patients with non‐hepatic abdominal malignancies. Although we excluded patients with overt cachexia, the potential impact of malignancy on systemic inflammation and muscle biology cannot be entirely ruled out. Furthermore, while strong associations were observed among C‐BAs, serum IL‐6 levels and skeletal muscle alterations, causality cannot be established due to the cross‐sectional design of this study. Elevated C‐BAs may reflect the severity of underlying liver dysfunction rather than acting as independent drivers of muscle pathology. Consequently, our findings are primarily exploratory, and future longitudinal or interventional studies are required to validate these relationships. Another point of uncertainty is the molecular mechanism by which tauro‐ and glyco‐C‐BAs directly act on skeletal muscle in patients with CLD.

**FIGURE 6:**
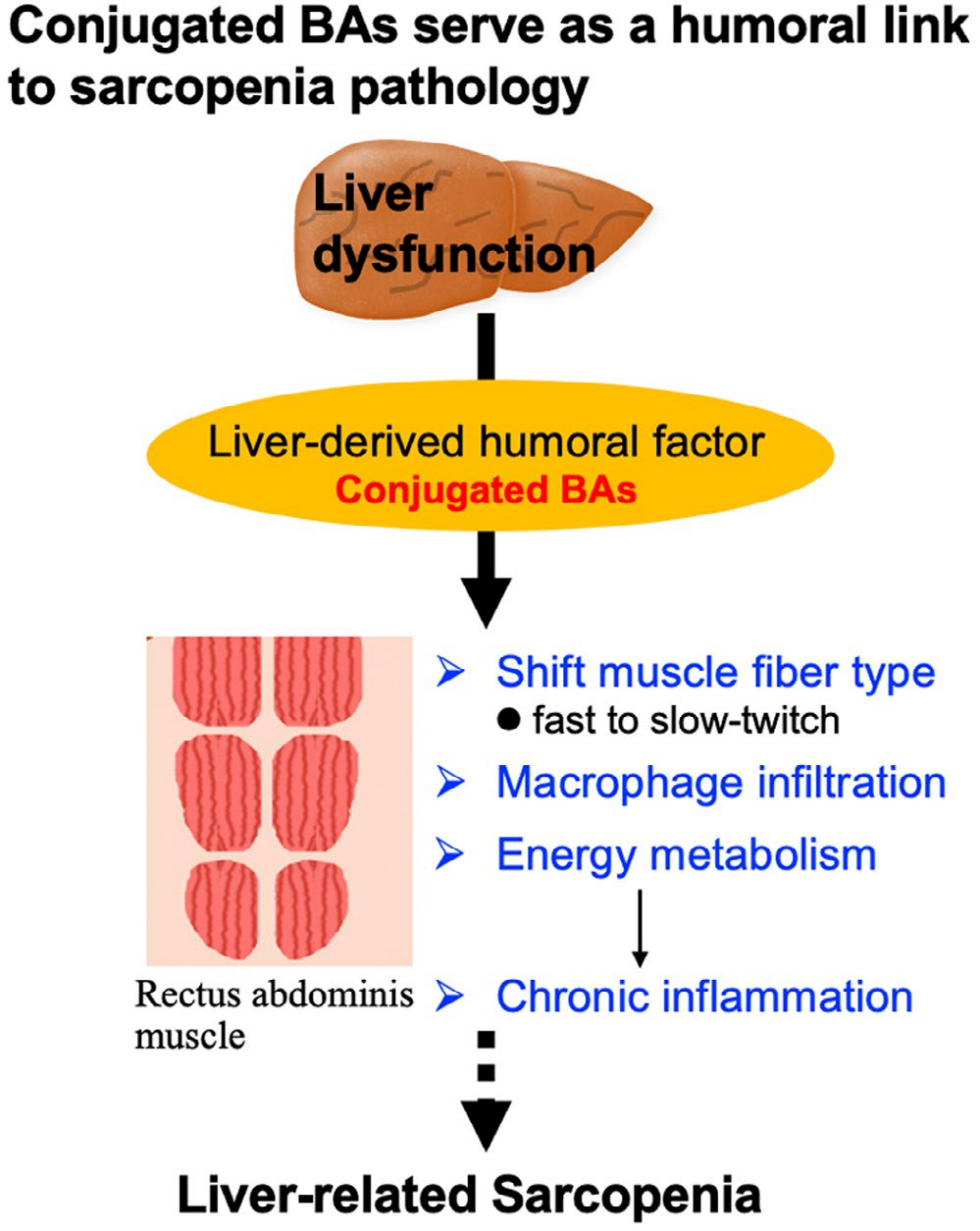
The graphical abstract of this study.

In conclusion, we have uncovered new roles for tauro‐ and glyco‐C‐BAs as negative regulators of skeletal muscle in patients with CLD. Serum IL‐6 may also have a modest contributory role in this effect. In CLD patients, elevated levels of C‐BAs lead to alterations in myosin fibre types and local chronic inflammation, suggesting their potential to induce a shift from fast to slow muscle fibres as well as muscle loss (Figure 6). The potential role of BA–lowering therapies, such as IBATs (IBAT), and IL‐6 receptor antagonists warrants further exploration. Tauro‐ and glyco‐C‐BAs − and possibly IL‐6 − may represent potential candidates as therapeutic targets for the prevention of sarcopenia in patients with CLD. However, this concept remains speculative at present, as it is based on associative findings and lacks direct support from interventional studies.

## Author Contributions

M.I., A.E., M.T. and K.I. performed most of the experiments, analysed the data and wrote the manuscript. M.K., Y.T., N.Y. and M.N. contributed to correct samples from patients. T.M., A.H. and T.I. contributed to measuring bile acids. A.E. and H.K. designed most of the experiments. Y.T., A.J.M., Y.K., H.N. and M.N. participated in the design, execution and analysis of the study. All authors have discussed the results from the experiments, read and approved the final version of the submitted manuscript.

## Funding

This work was supported by JSPS KAKENHI Grant Number 22K08011 and 25K11224 to M.I.

## Ethics Statement

This study protocol was reviewed and approved by the clinical research ethics review committee of NHO Kyushu Medical Center (Approval No. 10–29). In addition, the retrospective use of stored samples was approved by the clinical research ethics review committee of Mie University Hospital (Approval No. H2020‐035) and NHO Kyushu Medical Center (Approval No. 20C039).

## Consent

Informed written consent was obtained from all patients prior to enrollment. For the retrospective analysis of stored samples, an opt‐out approach was adopted, allowing patients to decline participation.

## Conflicts of Interest

The authors declare no conflicts of interest.

## Supporting information


**Table S1:** Oligonucleotides for mRNA expression.
**Table S2:** Demographic and Clinical Characteristics of HCC surgical patients. **Table S3:** Classification of the assessed Bile Acids.
**Table S4:** Bile acids and Characteristics of HCC surgical patient.
**Table S5:** Correlation between the ratio of conjugated to unconjugated BAs and laboratory variables.
**Figure S1:** Changes in total BA, primary BA, secondary BA, 12α‐OH BA and non‐12α‐OH BA levels among the three groups. BA, bile acid.
**Figure S2:** (A) Scatter plot between multifidus‐erector spinae muscle area and tauro‐conjugated BAs.
**Figure S3:** Scatter plot between chronic inflammation gene expression and clinical variables.

## Data Availability

The data that support the findings of this study are available on request from the corresponding author. The data are not publicly available due to privacy or ethical restrictions.
