# Supplementary material for: The Conjugated Bile Acids Profile Suggests a Novel Liver‐Muscle Axis Associated With Sarcopenia in Chronic Liver Disease
Source: Liver Int. 2026 Mar 28;46(5):e70612. doi: 10.1111/liv.70612 (PMC13032176; doi:10.1111/liv.70612)
Supplement: Supplementary file 1 — Table S1: Oligonucleotides for mRNA expression. Table S2: Demographic and Clinical Characteristics of HCC surgical patients. Table S3: Classification of the assessed Bile Acids. Table S4: Bile acids and Characteristics of HCC surgical patient. Table S5: Correlation between the ratio of conjugated to unconjugated BAs and laboratory variables. Figure S1: Changes in total BA, primary BA, secondary BA, 12α‐OH BA and non‐12α‐OH BA levels among the three groups. BA, bile acid. Figure S2: (A) Scatter plot between multifidus‐erector spinae muscle area and tauro‐conjugated BAs. Figure S3: Scatter plot between chronic inflammation gene expression and clinical variables. [file LIV-46-0-s001.docx]

**The Conjugated Bile Acids Profile Suggests a Novel Liver-Muscle Axis Associated with Sarcopenia in Chronic Liver Disease**

Motoh Iwasa^1, 2^, Akiko Eguchi^1, 3^, Motoyuki Kohjima^4^, Teruo Miyazaki^5^, Hiroshi Kitamura^6^, Yuko Takami^7^, Naoki Yamashita^4^, Mina Tempaku^1^, Kiyora Izuoka^1^, Yoshinao Kobayashi^1^, Yoshiyuki Takei^1^, Akira Honda^5^, Hayato Nakagawa^1^, Tadashi Ikegami^8^, Makoto Nakamuta^4^, Jun Okabe^9^ and Aldo J. Montano-Loza^10^

^1^ Department of Gastroenterology and Hepatology, Mie University Graduate School of Medicine, Tsu, Japan

^2^ Department of Gastroenterology, Murase Hospital, Suzuka, Japan

^3^ Biobank Center, Mie University Hospital, Tsu, Japan

^4^ Department of Gastroenterology, Clinical Research Institute, NHO Kyushu Medical Center, Fukuoka, Japan

^5^ Joint Research Center, Tokyo Medical University Ibaraki Medical Center, Inashiki-gun, Japan

^6^ Department of Laboratory Animal Medicine, Tohoku University School of Medicine, Sendai, Japan

^7^ Department of Hepato-Biliary-Pancreatic Surgery, NHO Kyushu Medical Center, Fukuoka, Japan

^8^ Department of Gastroenterology and Hepatology, Tokyo Medical University Ibaraki Medical Center, Inashiki-gun, Japan

^9^ Epigenetics in Human Health and Disease Laboratory, Baker Heart and Diabetes Institute, Melbourne, Victoria, Australia.

^10^ Division of Gastroenterology and Liver Unit, University of Alberta, Edmonton, Canada

**Corresponding author: Akiko Eguchi, Ph.D.**

2-174 Edobashi, Tsu, Mie, 514-8507, JAPAN

+81-59-231-9238

akieguchi@med.mie-u.ac.jp

**Supplementary Table 1. Oligonucleotides for mRNA expression**

| PAX7 | forward | ACCCCTGCCTAACCACATC |
| --- | --- | --- |
|  | reverse | GCGGCAAAGAATCTTGGAGAC |
| MYH2 | forward | AGAAACTTCGCATGGACCTAGA |
|  | reverse | CCAAGTGCCTGTTCATCTTCA |
| MYH7 | forward | CCCTACAAGTGGTTGCCAGTG |
|  | reverse | CTTCCCTGCGCCAGATTCTC |
| MYH1 | forward | ACTGCCGAGACCGAGTATG |
|  | reverse | GCGATCCTTGAGGTTGTAGAGC |
| MYH4 | forward | ACAAGGTTCTAAATGCGAGTGC |
|  | reverse | TGACCGAATTTGTACTGGGTG |
| PEPCK | forward | GGCTGAGAATACTGCCACACT |
|  | reverse | ACCGTCTTGCTCTCTACTCGT |
| SREBF1 | forward | CCATGGATTGCACTTTCGAA |
|  | reverse | GGCCAGGGAAGTCACTGTCTT |
| TFAM | forward | GTGGTTTTCATCTGTCTTGGCAAG |
|  | reverse | TTCCCTCCAACGCTGGGCAATT |
| IL6 | forward | ACTCACCTCTTCAGAACGAATTG |
|  | reverse | CCATCTTTGGAAGGTTCAGGTTG |
| ALF1 | forward | ATGAGCCAAACCAGGGATTTAC |
|  | reverse | GGGATCGTCTAGGAATTGCTTGT |
| CCL2 | forward | AGAATCACCAGCAGCAAGTGTCC |
|  | reverse | TCCTGAACCCACTTCTGCTTGG |
| TNF | forward | GAGGCCAAGCCCTGGTATG |
|  | reverse | CGGGCCGATTGATCTCAGC |
| AGER | forward | ACTACCGAGTCCGTGTCTACC |
|  | reverse | GGAACACCAGCCGTGAGTT |
| CD80 | forward | GGCCCGAGTACAAGAACCG |
|  | reverse | TCGTATGTGCCCTCGTCAGAT |
| SERPINE1 | forward | ACCGCAACGTGGTTTTCTCA |
|  | reverse | TTGAATCCCATAGCTGCTTGAAT |
| IL10 | forward | GACTTTAAGGGTTACCTGGGTTG |
|  | reverse | TCACATGCGCCTTGATGTCTG |
| CD209 | forward | AATGGCTGGAACGACGACAAA |
|  | reverse | CAGGAGGCTGCGGACTTTTT |
| CLEC10A | forward | AGCAACTTCACCTCAAACACTG |
|  | reverse | AGATGCTATCGTTTCTTCCAAGC |
| TGFB1 | forward | CAATTCCTGGCGATACCTCAG |
|  | reverse | GCACAACTCCGGTGACATCAA |
| β actin | forward | AAGTCCCTTGCCATCCTAAAA |
|  | reverse | ATGCTATCACCTCCCCTGTG |

**Supplementary Table 2. Demographic and Clinical Characteristics of HCC surgical patients**

| **Parameter** | **Chronic hepatitis**  **(N = 14)** | **Liver cirrhosis**  **(N = 22)** | **Non-CLD**  **(N = 6)** | **P-values** |
| --- | --- | --- | --- | --- |
| Age (yr) | 79.5 (74.5, 81.3) | 74 (66.8, 78.0) | 65.5 (56.5, 73.5) | P(G1-G2) =0.015  P (G1-G3) = 0.018  P (G2-G3) = 0.136 |
| Sex (M), n (%) | 11 (78.6%) | 6 (27.3%) | 4 (66.7%) | P (G1-G2) = 0.005  P (G1-G3) = 0.613  P (G2-G3) = 0.147 |
| BMI (kg/m^2^) | 22.1 (19.9, 25.5) | 23.8 (21.5, 26.7) | 20.6 (18.3, 21.5) | P (G1-G2) = 0.284  P (G1-G3) = 0.187  P (G2-G3) = 0.007 |
| Etiology, n (%)   - HBV - HCV - Colon Ca meta - Brest Ca meta - Pancreatic Ca meta - GB Ca meta - CBD Ca meta | 3 (21.4%)  11 (78.6%)  0  0  0  0  0 | 2 (9.1%)  20 (90.9%)  0  0  0  0  0 | 0  0  1 (16.7%)  2 (33.3%)  1 (16.7%)  1 (16.7%)  1 (16.7%) | P <0.001 |
| AST (U/L) | 37.0 (24.8, 43.0) | 61.5 (48.8, 81.0) | 24.0 (19.5, 38.3) | P (G1-G2) = <0.001  P (G1-G3) = 0.090  P (G2-G3) = <0.001 |
| ALT (U/L) | 33.0 (17.5, 46.3) | 44.5 (34.8, 63.8) | 22.0 (18.0,53.3) | P (G1-G2) = 0.032  P (G1-G3) = 0.934  P (G2-G3) =0.145 |
| γ-GT (U/L) | 37.0 (26.5 66.8) | 55.5 (27.0, 86.0) | 40.5 (25.5, 115.5) | P (G1-G2) = 0.427  P (G1-G3) = 0.904  P (G2-G3) = 0.713 |
| ALP | 304.0 (250.3, 348.5) | 394.0 (349.0, 540.5) | 418.5 (292.5, 487.3) | P (G1-G2) = 0.005  P (G1-G3) = 0.117  P (G2-G3) =0.955 |
| ALB (g/dL) | 4.1 (3.6, 4.4) | 3.5 (3.2, 3.9) | 4.0 (3.5, 4.3) | P (G1-G2) = 0.001  P (G1-G3) = 0.430  P (G2-G3) =0.063 |
| T-Bil (mg/dL) | 0.6 (0.5, 0.7) | 0.9 (0.6, 1.2) | 0.6 (0.5, 0.8) | P (G1-G2) = 0.004  P (G1-G3) = 0.900  P (G2-G3) =0.023 |
| ALBI score | -2.8 (-0.3, -2.6) | -2.2 (-2.4, -1.9) | -2.8 (-3.0, -2.2) | P(G1-G2) <0.001  P (G1-G3) = 0.458  P (G2-G3) =0.022 |
| PT (%) | 90.5 (86.0, 93.5) | 87.0 (83.5, 89.0) | 96.5 (90.3, 110.3) | P (G1-G2) = 0.115  P (G1-G3) = 0.057  P (G2-G3) =0.009 |
| Glucose | 95.0 (84.0, 108.0) | 99.0 (92.8, 117.5) | 103.0 (93.5, 144.0) | P (G1-G2) = 0.063  P (G1-G3) = 0.091  P (G2-G3) =0.574 |
| HbA1c | 5.1 (4.8, 5.3) | 5.3 (5.0, 6.0) | 6.7 (5.8, 6.9) | P (G1-G2) = 0.210  P (G1-G3) =0.026  P (G2-G3) =0.026 |
| IL-6 | 3.5 (2.7, 4.8) | 5.4 (3.2, 8.2) | 4.1 (2.5, 13.8) | P (G1-G2) = 0.105  P (G1-G3) = 0.621  P (G2-G3) =0.634 |
| Myostatin | 0.06 (0.04, 0.11) | 0.04 (0.02, 0.06) | 0.03 (0.02, 0.05) | P (G1-G2) = 0.029  P (G1-G3) = 0.096  P (G2-G3) =1.000 |
| IGF-1 | 0.01 (0.01, 0.02) | 0.02 (0.01, 0.03) | 0.01(0.01, 0.1) | P (G1-G2) = 0.099  P (G1-G3) = 0.930  P (G2-G3) =0.243 |
| PLT (x10^4^ cells/μL) | 14.1 (11.7, 18.0) | 10.0 (8.1, 14.4) | 20.8 (15.6, 27.6) | P (G1-G2) = 0.019  P (G1-G3) = 0.026  P (G2-G3) =0.004 |
| Psoas muscle area  (cm^2^) | 18.35 (15.28, 23.25) | 17.50 (14.58, 20.33) | 20.90 (15.85, 22.00) | P (G1-G2) = 0.311  P (G1-G3) = 0.823  P (G2-G3) = 0.232 |
| Psoas muscle index  (cm^2^/m^2^) | 7.34 (6.64, 8.81) | 7.27 (6.24, 8.04) | 7.79 (5.85, 9.24) | P (G1-G2) = 0.689  P (G1-G3) = 0.754  P (G2-G3) = 0.606 |
| Multifidus-erector spinae muscle area (cm^2^) | 32.75 (28.70, 41.43) | 29.95 (26.45, 36.33) | 42.10 (35.35, 43.05) | P (G1-G2) = 0.311  P (G1-G3) = 0.186  P (G2-G3) = 0.023 |
| Multifidus-erector spinae muscle index (cm^2^/m^2^) | 7.34 (6.64, 8.81) | 7.26 (6.24, 8.04) | 7.79 (5.85, 9.24) | P (G1-G2) = 0.962  P (G1-G3) = 0.257  P (G2-G3) = 0.113 |

The comparisons between groups performed using the Whitney U test for continuous variables. Chi-square test was used for categorical variables. Data reported as Median (25^th^ ,75^th^ percentile). p value <0.05 is significant. Group 1 (G1): Chronic hepatitis, Group 2 (G2): Liver cirrhosis, Group 3 (G3): Non-CLD

BMI, body mass index; HBV, hepatitis B virus; HCV, hepatitis C virus; Ca, cancer; meta, metastasis; GB, gall bladder; CBD, common bile duct; AST, aspartate aminotransferase; ALT, alanine aminotransferase; γ-GT, glutamyl-transferase; ALP, alkaline phosphatase; ALB, albumin; T-Bil, total bilirubin; ALBI, albumin-bilirubin; PT, prothrombin time; HbA1c, glycated hemoglobin A1c; IL, interleukin; IGF-1, insulin growth factor-1; PLT, platelet count.

Statistics include number (%) or median (25th, 75th percentile).

**Supplementary Table 3. Classification of the assessed Bile Acids**

| **Type of Bile Acid** | **Name** |
| --- | --- |
| Primary bile acids | Cholic acid (CA)  Chenodeoxycholic acid (CDCA) |
| Secondary Bile Acids | Deoxycholic acid (DCA)  Lithocholic acids (LCA) |
| 12α-OH Bile Acids | CA, Tauro-conjugated CA, Glyco-conjugated CA,  DCA, Tauro-conjugated DCA, Glyco-conjugated DCA |
| Non-12α-OH Bile Acids | CDCA, Tauro-conjugated CDCA, Glyco-conjugated CDCA  LCA, Tauro-conjugated LCA, Glyco-conjugated LCA  UDCA, Tauro-conjugaed UDCA, Glyco-conjugated UDCA |

**Supplementary Table 4. Bile acids and Characteristics of HCC surgical patient**

| **Parameter** | **Chronic hepatitis**  **(N = 14)** | **Liver cirrhosis**  **(N = 22)** | **Non-CLD**  **(N = 6)** | **P-values** |
| --- | --- | --- | --- | --- |
| Total bile acids | 14.4 (5.1, 22.0) | 30.7 (17.0, 63.1) | 4.1 (1.7, 5.8) | P (G1-G2) = 0.010  P (G1-G3) = 0.068  P (G2-G3) <0.001 |
| Primary BA | 4.52 (3.29, 7.39) | 13.91 (6.52, 22.45) | 2.39 (0.59, 3.42) | P (G1-G2) = 0.003  P (G1-G3) = 0.128  P (G2-G3) <0.001 |
| Secondary BA | 7.11 (1.95, 14.82) | 18.89 (5.62, 33.53) | 1.20 (0.80, 2.82) | P (G1-G2) = 0.116  P (G1-G3) = 0.071  P (G2-G3) = 0.002 |
| 12α-OH BA | 2.81 (1.08, 3.95) | 4.61 (3.39, 7.33) | 1.60 (0.93, 2.55) | P (G1-G2) = 0.007  P (G1-G3) = 0.431  P (G2-G3) = 0.004 |
| Non-12α-OH BA | 10.66 (3.11, 18.81) | 24.62 (10.73, 55.51) | 2.05 (0.43, 3.59) | P (G1-G2) = 0.027  P (G1-G3) = 0.045  P (G2-G3) <0.001 |
| Chenodeoxycholic acid (CDCA) | 0.76 (0.26, 0.99) | 0.42 (0.30, 1.52) | 0.24 (0.12, 0.81) | P (G1-G2) = 0.662  P (G1-G3) = 0.121  P (G2-G3) = 0.187 |
| Tauro-conjugated CDCA (TCDCA) | 0.35 (0.03, 1.55) | 2.11 (1.08, 5.89) | 0.03 (0.003, 0.21) | P (G1-G2) = 0.005  P (G1-G3) = 0.113  P (G2-G3) <0.001 |
| Glyco-conjugated CDCA (GCDCA) | 2.22 (1.33, 4.24) | 6.55 (3.19, 8.83) | 1.21 (0.26, 1.97) | P (G1-G2) = 0.002  P (G1-G3) = 0.254  P (G2-G3) <0.001 |
| Colic acid (CA) | 0.26 (0.16, 0.39) | 0.18 (0.11, 0.42) | 0.11 (0.09, 0.28) | P (G1-G2) = 0.702  P (G1-G3) = 0.091  P (G2-G3) = 0.132 |
| Tauro-conjugated CA (TCA) | 0.01 (0.003, 0.04) | 0.35 (0.08, 0.70) | 0.002 (0.0004, 0.006) | P (G1-G2) <0.001  P (G1-G3) = 0.128  P (G2-G3) <0.0001 |
| Glyco-conjugated CA (GCA) | 0.57 (0.21, 1.05) | 2.46 (1.16, 3.40) | 0.21 (0.12, 0.38) | P (G1-G2) <0.001  P (G1-G3) = 0.236  P (G2-G3) <0.001 |
| Deoxycholic acid (DCA) | 0.37 (0.13, 0.89) | 0.29 (0.09, 0.51) | 0.53 (0.14, 1.10) | P (G1-G2) = 0.287  P (G1-G3) = 0.949  P (G2-G3) = 0.391 |
| Tauro-conjugated DCA (TDCA) | 0.03 (0.005, 0.19) | 0.14 (0.02, 0.47) | 0.02 (0.003, 0.03) | P (G1-G2) = 0.078  P (G1-G3) = 0.233  P (G2-G3) = 0.010 |
| Glyco-conjugated DCA (GDCA) | 0.79 (0.07, 1.77) | 1.48 (0.47, 2.38) | 0.66 (0.33, 1.08) | P (G1-G2) = 0.126  P (G1-G3) = 0.799  P (G2-G3) = 0.160 |
| Lithocholic acids (LCA) | 0.04 (0.02, 0.12) | 0.06 (0.02, 0.22) | 0.03 (0.01, 0.04) | P (G1-G2) = 0.898  P (G1-G3) = 0.279  P (G2-G3) = 0.214 |
| Tauro-conjugated LCA (TLCA) | 0.00005 (0.0001, 0.001) | 0.0004 (0.0002, 0.003) | 0.0002 (0.0002, 0.001) | P (G1-G2) = 0.860  P (G1-G3) = 0.681  P (G2-G3) = 0.571 |
| Glyco-conjugated LCA (GLCA) | 0.003 (0.0002, 0.06) | 0.004 (0.0002, 0.10) | 0.002 (0.0002, 0.005) | P (G1-G2) = 0.666  P (G1-G3) = 0.489  P (G2-G3) = 0.292 |
| TCDCA/CDCA ratio | 0.43 (0.09, 1.40) | 5.23 (1.59, 11.46) | 0.07 (0.05, 0.65) | P (G1-G2) = 0.001  P (G1-G3) = 0.271  P (G2-G3) < 0.001 |
| GCDCA/CDCA ratio | 3.54 (1.25, 6.89) | 11.25 (6.33, 18.09) | 4.60 (1.56, 8.65) | P (G1-G2) = 0.002  P (G1-G3) = 0.933  P (G2-G3) = 0.024 |
| TCA/CA | 0.03 (0.01, 0.23) | 1.35 (0.27, 4.62) | 0.02 (0.004, 0.05) | P (G1-G2) < 0.001  P (G1-G3) = 0.330  P (G2-G3) < 0.001 |
| GCA/CA | 1.44 (0.75, 4.53) | 8.89 (3.33, 20.0) | 1.81 (0.40, 2.69) | P (G1-G2) = 0.001  P (G1-G3) = 0.679  P (G2-G3) = 0.004 |
| TDCA/DCA | 0.10 (0.03, 0.24) | 0.63 (0.15, 1.45) | 0.02 (0.01, 0.26) | P (G1-G2) = 0.005  P (G1-G3) = 0.360  P (G2-G3) = 0.002 |
| GDCA/DCA | 1.11 (0.68, 2.74) | 4.90 (2.80, 7.02) | 0.86 (0.62, 7.83) | P (G1-G2) = 0.001  P (G1-G3) = 0.808  P (G2-G3) = 0.033 |
| TLCA/LCA | 0.009 (0.004, 0.04) | 0.009 (0.006, 0.03) | 0.01 (0.004, 0.06) | P (G1-G2) = 0.995  P (G1-G3) = 0.946  P (G2-G3) = 0.947 |
| GLCA/LCA | 0.06 (0.01, 0.42) | 0.15 (0.009, 0.45) | 0.05 (0.007, 0.23) | P (G1-G2) = 0.552  P (G1-G3) = 0.600  P (G2-G3) = 0.319 |

The pair-wised comparisons between groups performed using Mann Whitney U test. Data reported as Median (25^th^, 75^th^ percentile). p value <0.05 is significant. Group 1 (G1): Chronic hepatitis, Group 2 (G2): Liver cirrhosis, Group 3 (G3): Non-CLD

**Supplementary Table 5. Correlation between the ratio of conjugated to unconjugated BAs and laboratory variables.**

**BA** **AST (U/L) ALB (g/dL) T-Bil (mg/dL) ALBI score PT (%) PLT(X10^4^ cells/uL) ALT (U/L) γ-GT (U/L)**

Sr Sr Sr Sr Sr Sr Sr Sr

P value P value P value  P value P value P value P value P value

**TCDCA/CDCA**

0.438 -0.429 0.369 0.512 -0.340 -0.387

<0.01 <0.01 <0.05 <0.001 <0.05 <0.05

**GCDCA/CDCA**

0.366 0.441 -0.434 0.360 0.330

<0.05 <0.01 <0.01 <0.05 <0.05

**TCA/CA** 0.463 -0.411 0.351 0.496 -0.389 -0.406

<0.01 <0.01 <0.05 <0.001 <0.05 <0.01

**GCA/CA** 0.367 -0.314 0.382 0.381 -0.363 -0.367

<0.05 <0.05 <0.05 <0.05 <0.05 <0.05

**TDCA/DCA**

0.463 -0.515 0.379 0.586 -0.475 -0.307

<0.01 <0.001 <0.05 <0.0001 <0.01 <0.05

**GDCA/DCA**

0.518 -0.308 0.565 0.412 -0.389 -0.375

<0.001 <0.05 <0.0001 <0.01 <0.05 <0.05

AST, aspartate aminotransferase; ALT, alanine aminotransferase; γ-GT, glutamyl-transferase; ALP, alkaline phosphatase; ALB, albumin; T-Bil, total bilirubin; ALBI, albumin-bilirubin; PT, prothrombin time; PLT, platelet count; Sr: spearman r; BA, bile acid; CA, cholic acid; CDCA, chenodeoxycholic acid; DCA, deoxycholic acid.

**Supplementary Figure 1**

**
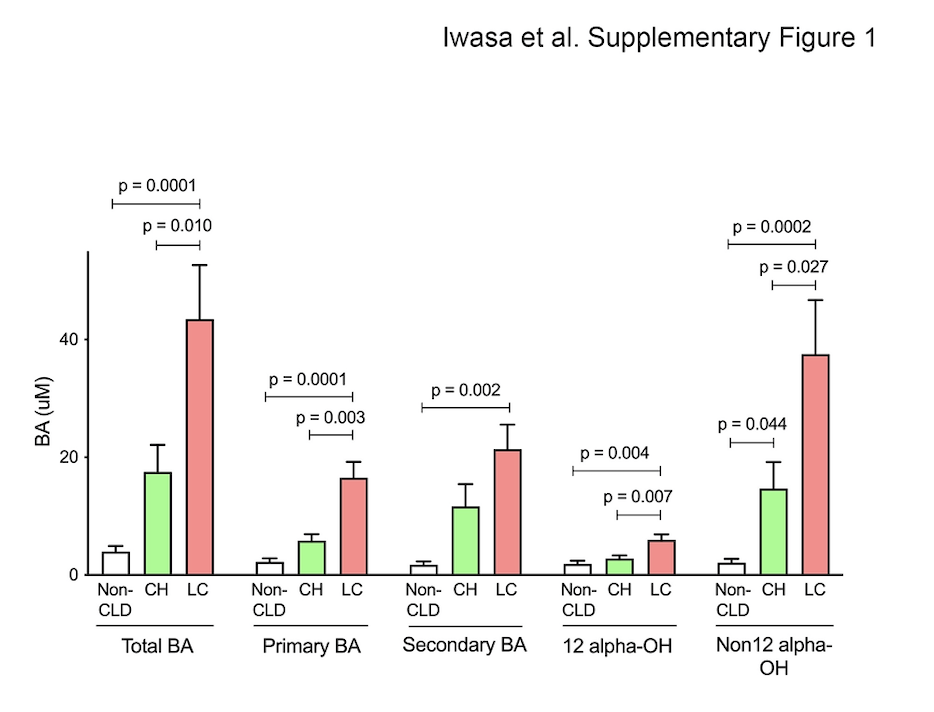
**

Changes in total BA, primary BA, secondary BA, 12α-OH BA, and non-12α-OH BA levels among the three groups.

BA, bile acid.

**Supplementary Figure 2**

**
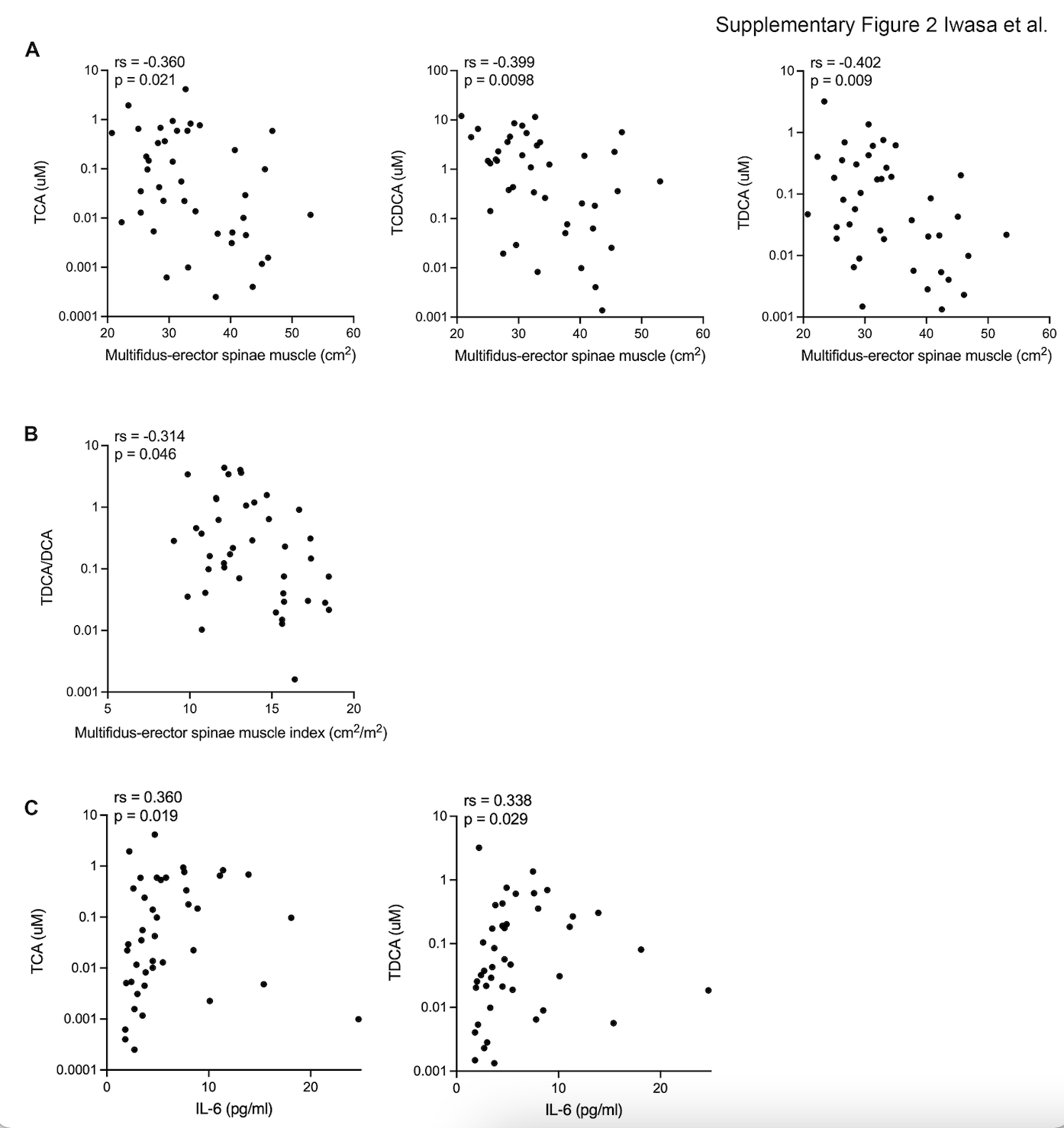
**

**(A)** Scatter plot between multifidus-erector spinae muscle area and tauro-conjugated BAs. **(B)** Scatter plot between multifidus-erector spinae muscle index and tauro-conjugated BAs. **(C)** Scatter plot between IL-6 and tauro-conjugated BAs.

BA, bile acid; CDCA, chenodeoxycholic acid; CA, cholic acid, DCA, deoxycholic acid, IL-6, interleukin-6.

**Supplementary Figure 3**

**
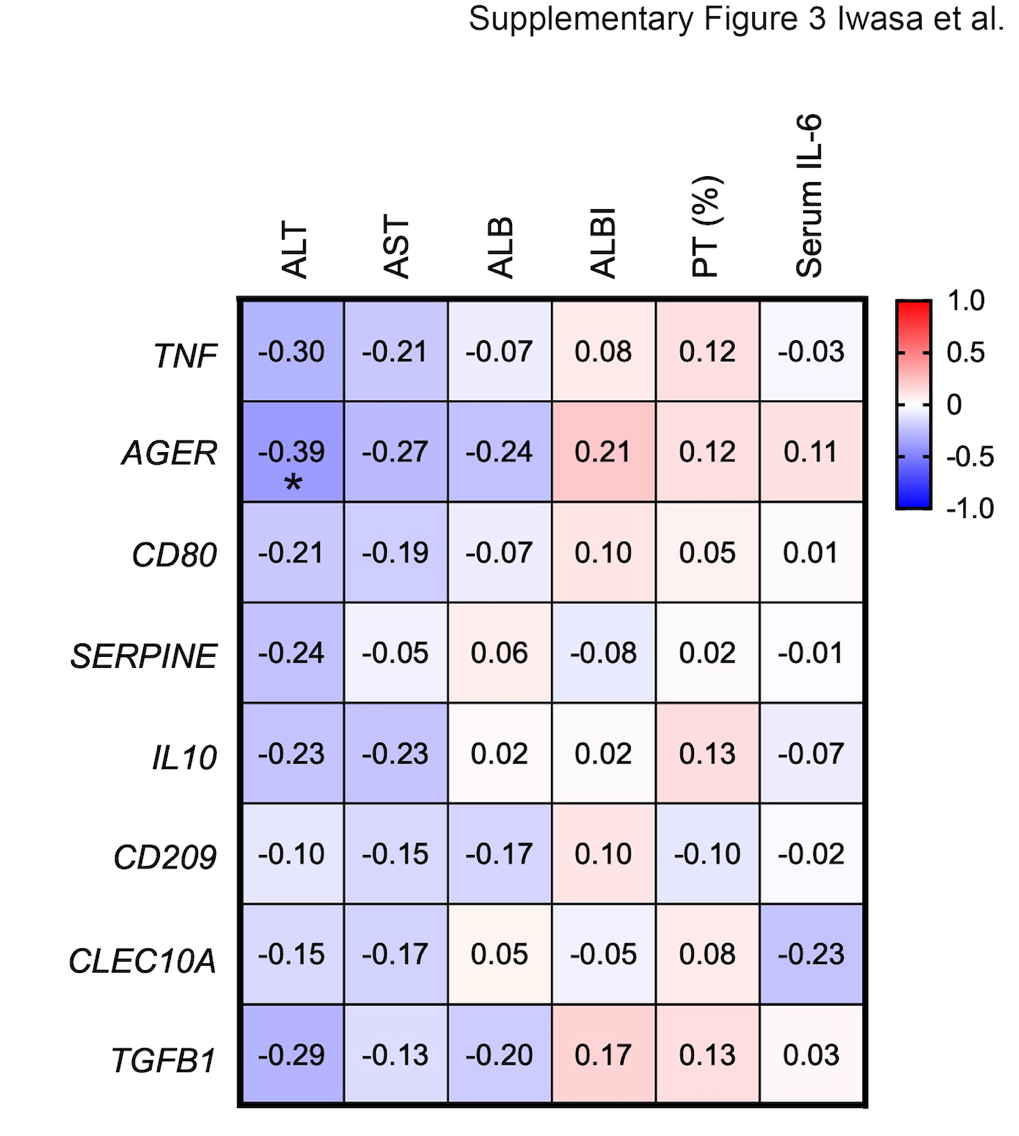
**

Scatter plot between chronic inflammation gene expression and clinical variables.
